# Enhancing the Quality of Traditional Indonesian Shrimp Paste (*Terasi*) Through *Tetragenococcus halophilus* 54M106-3 Inoculation: Physicochemical, Sensory, and Bioactivity Insights

**DOI:** 10.3390/foods14142419

**Published:** 2025-07-09

**Authors:** Muhammad Alfid Kurnianto, Safrina Isnaini Adirama, Wenxi Xu, Sri Winarti, Dina Mustika Rini

**Affiliations:** 1 Food Technology Study Program, Faculty of Engineering and Science, Universitas Pembangunan Nasional Veteran Jawa Timur, Surabaya 60294, Indonesia; m.alfid.tp@upnjatim.ac.id (M.A.K.);; 2 College of Food Science and Technology, Wuhan Business University, Wuhan 430056, China; 20241164@wbu.edu.cn; 3 Graduate School of Integrated Sciences for Life, Hiroshima University, 1-4-4 Kagamiyama, Higashi-Hiroshima 739-8528, Japan

**Keywords:** shrimp, *terasi*, traditional food, fermentation, functional food, starter culture

## Abstract

*Terasi* is a traditional Indonesian fermented condiment made from rebon shrimp and salt. This study investigated the effects of *Tetragenococcus halophilus* inoculation and varying salt concentrations (6%, 12%, and 18%) on the physicochemical and sensory properties of *terasi*, compared to a non-inoculated control (25% salt), after 7, 14, and 21 days of fermentation. Inoculation decreased pH, soluble protein, and texture while increasing N-amino acid content, moisture, lactic acid bacteria (LAB), and color darkening. Higher salt levels raised pH, soluble protein, and texture but reduced N-amino acids, moisture, and LAB, resulting in a lighter color. LAB activity peaked on day 7, with moisture and texture increasing over time. Sensory analysis favored inoculated samples, and TOPSIS identified *terasi* with *T. halophilus*, 6% salt, and 7 days of fermentation as optimal in quality and preference. This formulation also demonstrated strong bioactivity, including antioxidant activity (3.90 mg AEAC/g sample by DPPH assay and 8.76 ± 0.22 mg AEAC/g sample by FRAP assay), antidiabetic potential via α-amylase and α-glucosidase inhibition (IC_50_ of 1.95 and 7.24 mg/mL), and antimicrobial effects against *E. coli* (32.78 mm) and *S. aureus* (30.85 mm). These results suggest that *T. halophilus*-inoculated *terasi* offers enhanced quality and functional properties, supporting its potential as a health-promoting fermented food product.

## 1. Introduction

*Terasi*, a traditional condiment in Indonesia, is a fermented product traditionally made by combining crushed planktonic shrimp (*rebon*) with salt [1]. Similar products to *terasi* are found in various Asian countries under different names, with variations in flavor, color, and texture influenced by raw materials, processing methods, culinary practices, consumer preferences, and regional climatic conditions [2]. *Terasi* is characterized by a coarse texture, strong aroma, and savory taste. *Terasi* is primarily produced through traditional techniques involving salting, grinding, drying, and fermentation [1].

The fermentation process is critical for developing the organoleptic characteristics of *terasi*, with longer fermentation times enhancing its flavor. This predominantly spontaneous fermentation process highlights the critical role of salt in shaping microbial diversity, which significantly impacts the overall integrity and hygiene of the final product [3]. Moreover, challenges associated with the fermentation process necessitate control measures, such as the use of competitive starter cultures, such as *Tetragenococcus halophilus.*

*T. halophilus*, a salt-tolerant lactic acid bacterium (LAB), is frequently present in a variety of fermented foods, such as fish sauce, shrimp paste, and fermented black beans [4,5,6]. This species thrives in high-salinity environments and tolerates NaCl concentrations above 18% [7]. The use of *T. halophilus* as a starter culture in fermentation processes has been well documented. For instance, Yu et al. [5] reported that inoculating *T. halophilus* at a concentration of 10^7^ CFU/mL in Chinese shrimp paste fermentation notably shortened the fermentation period while enhancing organoleptic properties such as texture, appearance, and overall quality. Volatile compound analysis using HS-GC-IMS/MS revealed that the volatile fingerprint profile of the resulting product closely resembled that of traditional Chinese shrimp paste [5]. Similarly, Udomsil et al. [8] reported that *T. halophilus* enhanced the flavor of fermented fish sauce by improving amino acid and volatile compound profiles and reducing biogenic amine content.

Therefore, incorporating a halophilic starter in *terasi* production is expected to influence the fermentation rate, physicochemical properties, and biological activity of the product. To date, no study has explored the use of *T. halophilus* as a starter culture to enhance the quality of *rebon* shrimp (*Acetes erythraeus*) *terasi* and identified the potential bioactivity derived from its application in *terasi*. Therefore, this study aimed to examine the effects of *T. halophilus* and various salt concentrations on improving the quality of *rebon* shrimp *terasi*. The optimal conditions were further evaluated for biological activity.

## 2. Materials and Methods

### 2.1. Culture Starter Preparation

Culture starters were prepared following the methods described by Todorov et al. [9]. Briefly, 0.1 mL of bacterial culture of *T. halophilus* 54M106-3 from the Indonesian Culture Collection was cultured in De Man, Rogosa, and Sharpe (MRS) broth (Oxoid, Basingstoke, UK). The starters were then incubated at 37 °C for 24 h.

### 2.2. Terasi Preparation

*Terasi* shrimp paste was prepared following a previously described method [10]. The *rebon* (*Acetes erythraeus*) shrimp were washed and dried in a cabinet dryer at 60 °C for 3–4 h. The *rebon* shrimp were then ground in a half-dried condition. The partially dried shrimp were then ground and mixed with varying salt concentrations (6%, 12%, and 18%). The mixtures were subsequently inoculated with *T. halophilus*, with each inoculum added at a density of 10^8^ CFU/mL before fermentation. Fermentation was carried out in sealed jars at 37 °C for 0, 7, 14, and 21 days. Following fermentation, the samples were dried again in a cabinet dryer at 60 °C for 3 to 4 h.

### 2.3. Determination of Color and Moisture Content

The color of the *terasi* was measured using an IWAFE colorimeter WF30 (8 mm) (IWAVE, Shanghai, China) and was recorded in terms of L* for lightness, a* for redness/greenness, and b* for yellowness/blueness. Furthermore, the total color differences (Δ*E*) were compared to the control sample (non-inoculated *terasi* with 25% salt addition at day 0 of the fermentation stage) using Equation (1) [11].

(1) $$∆E=\sqrt{{\left(L^{*}-L_{0}^{*}\right)}^{2}+{\left(a^{*}-a_{0}^{*}\right)}^{2}+{\left(b^{*}-b_{0}^{*}\right)}^{2}}$$

Meanwhile, the chroma was calculated according to Equation (2) [12] and then further compared with the control sample to obtain the total chroma difference.

(2) $$C^{*}=\sqrt{a^{*2}+b^{*2}}$$

Moisture content was determined using the gravimetric method by oven-drying the samples at 100–102 °C for 6 h.

### 2.4. Determination of pH

The pH was determined using a pH meter (Suntex, Taiwan, China) after calibration with standard buffers at pH 4.0 and 7.0. Before measurement, the *terasi* samples were diluted 1:10 (*w*/*v*) in distilled water according to the previously described method [13].

### 2.5. Determination of Texture

The texture of *terasi* was analyzed using a texture profile analysis with a Texture Analyzer TVT 6700 (Evalab, Adana, Turkey). The samples (size 3 × 3 × 3 cm^3^) were pressed twice with a probe (6 mm diameter). The speed of the probe was set to 5 mm/s, and the sample was pressed to 30% of its initial height. The parameters observed were hardness, using the macro program of the TexCalc 5 version 5.2.5.336 software.

### 2.6. Determination of Soluble Protein

The soluble protein content was measured using the Lowry method with slight modifications, as described previously [14]. Lowry reagent C was formulated by mixing 50 mL of reagent A (2% Na_2_CO_3_ in 0.1 M NaOH) with 1 mL of reagent B (0.5% CuSO_4_-5H_2_O in 1% potassium tartrate). The samples (10 g) were ground and dissolved in 100 mL of distilled water. For analysis, 1 mL of either the sample or standard solution was combined with 5 mL of reagent C in a test tube, vortexed thoroughly, and incubated at room temperature for 15 min. Three drops of Reagent E (Folin–Ciocalteu reagent, Sigma-Aldrich, Darmstadt, Germany) were added, vortexed, and incubated for another 30 min at room temperature. Protein content was quantified by measuring the absorbance of the reaction mixture at its maximum stability in the wavelength range of 450–610 nm using a spectrophotometer (Thermo Fisher, Chesire, UK).

### 2.7. Determination of N-Amino Acid Content

N-amino content Nitrogen was analyzed using the formol titration method [15], as previously described, with slight modifications. A 10 g sample was ground, dissolved in 100 mL of distilled water, and then filtered using Whatman No. 1 filter paper (Whatman, Maidstone, UK). The resulting filtrate was adjusted to a final volume of 100 mL using distilled water. Next, 10 mL of the sample solution was combined with 20 mL of distilled water, 0.4 mL of saturated potassium oxalate, and a single drop of 1% phenolphthalein (PP) indicator solution. The mixture was then stirred and allowed to stand for 2 min. The reaction mixture was then titrated with 0.1 N NaOH until it turned pink. Two drops of 40% formaldehyde and a PP indicator were added to the titrated sample, followed by a second titration with 0.1 N NaOH. The N-amino acid content was calculated using Equation (3).

(3) $$N\left(\%\right)=\left(\frac{a}{b\times 10}\right)\times N\;NaOH\times Ar\;N\times Df$$

Whereas *a* is the formal titration volume, *b* is the sample weight, and *Df* is the diluent factor.

### 2.8. Determination of Total Lactic Acid Bacteria

LAB analysis was performed by total plate count, as previously described, with minor modification [13]. Serial dilution was performed by homogenizing 10 g of the sample in 90 mL of sterile physiological saline, resulting in a 10^−1^ dilution. Further serial dilutions were performed, and 1 mL aliquots of the 10^−7^, 10^−8^, and 10^−9^ dilutions were transferred to sterile Petri dishes. MRSA medium (Oxoid, Basingstoke, UK) was poured onto each plate and allowed to solidify. The plates were stored at 37 °C for 48 h. Colony counts were performed when the number of colonies formed after incubation ranged from 25 to 250. The number of colonies was counted and expressed as colony-forming units per milliliter (CFU/mL).

### 2.9. Sensory Evaluation

A hedonic test was conducted with a panel of 25 untrained undergraduate students from the Department of Food Technology, Universitas Pembangunan Nasional “Veteran” Jawa Timur, as previously described [16]. The sensory attributes evaluated included aroma, color, and texture. *Terasi* samples were prepared and presented at room temperature, each coded with a unique three-digit identifier for the panelists. The samples were evaluated in a randomized order to minimize potential order effects. The panelists rated the sensory attributes on a five-point hedonic scale, with 1 indicating “most disliked” and 5 indicating “most liked.” To maintain a controlled and unbiased evaluation environment, the panelists evaluated the samples individually at isolated stations. Drinking water was provided to cleanse the palate between the evaluations to ensure the reliability of the sensory evaluations.

### 2.10. Determination of Optimal Treatment

The Technique for Order of Preference by Similarity to Ideal Solution (TOPSIS) was used to select the best treatment based on physicochemical and sensory attributes. Following the approach described by Hedayati et al. [17], the process involved constructing a decision matrix, where salt concentration and fermentation time were alternatives, and attributes such as color (L*, a*, and b* values), texture, moisture content, N-amino acid, soluble protein, pH, total LAB, and sensory attributes (color, aroma, and texture) served as the criteria. The matrix was normalized and weighted using the best–worst multi-criteria decision-making method [18] and then used to calculate positive and negative ideal solutions. The distance of the alternatives from these solutions was determined, and the relative closeness index (cc_i_) was calculated, with higher values indicating more favorable formulations [17].

### 2.11. Amino Acid Analysis

Amino acid quantification was performed using high-performance liquid chromatography (HPLC) according to a modified method described by Afifah et al. [19]. Analytes were separated using an Agilent 1200 Series HPLC (Santa Clara, CA, USA) with an XDC-C-18 column (4.60 × 150 mm; 5 μm) and a photodiode array detector. The analysis process used an elution gradient with mobile phase A of 10% acetonitrile (*v*/*v*) and mobile phase B of 50% water (*v*/*v*), running at a flow rate of 0.5 mL/min for 30 min. The analytical column was thermostatted at 49 °C.

Protein hydrolysis was performed by adding 1 mL of 6 N HCl to 3 mg of protein samples in a vacuum-sealed ampoule. The mixtures were frozen using a dry ice-acetone immersion bath and then lyophilized using a vacuum pump. After removing the air, the vials were vacuum-sealed for 20 min and sealed by heating over a flame. The sealed ampoule was then placed in an incubator at 110 °C for 24 h to hydrolyze the samples. After hydrolysis, the sample was allowed to cool to ambient temperature, and the ampoule was rinsed with 2 mL of 0.01 N HCl. The rinsing liquid was transferred to an evaporation flask, and this step was repeated 2–3 times. The samples were freeze-dried under vacuum, rehydrated with 10–20 mL of water, and freeze-dried again 2–3 times to convert cysteine to cystine. Finally, 5 mL of HCl was added to the dried sample to prepare it for analysis.

For reagent preparation, ortho-phthalaldehyde (OPA) reagents were produced by dissolving 50 mg of OPA in 4 mL of methanol and subsequently adding mercaptoethanol, 30% Brij solution, and borate buffer. This solution was stored in a dark bottle at 4 °C, where it remained stable for one week. The derivatization reagent was freshly prepared daily by mixing one part of the OPA stock solution with two parts of the potassium borate buffer (pH 10.4). For amino acid analysis, the hydrolyzed samples were dissolved in 5 mL of HCl (0.01 N) and then filtered using Millipore paper. An equal volume of potassium borate buffer (pH 10.5) was then added. A 10 μL aliquot of the prepared sample was transferred into a clean ampoule, followed by the addition of 25 μL OPA reagent. The mixture was allowed to react for 1 min to ensure complete derivatization. Subsequently, a 5 μL portion of the sample was injected into the HPLC column for amino acid separation and analysis.

### 2.12. Antioxidant Activity Evaluation

The antioxidant capacities of the samples were examined by the 2,2-diphenyl-1-picrylhydrazyl (DPPH) and ferric reducing antioxidant power (FRAP) methods, as previously described [20,21]. For the DPPH assay, 2 mL of DPPH solution (prepared in methanol) was combined with 2 mL of either the sample or a standard solution (ascorbic acid) in the absence of light. The mixtures were then incubated at ambient temperature for 30 min in the dark. After incubation, the optical density of the solution was measured at 517 nm using a UV-Vis spectrophotometer (Genesys 10S UV-VIS, Thermo Fisher, Chesire, UK) with ethanol as the blank. Antioxidant activity was expressed as mg ascorbic acid equivalent antioxidant capacity (mg AEAC/g).

For the FRAP assay, stock reagents, including 2.5 mL of 100 mM TPTZ solution in 40 mM HCL, 25 mL of 300 mM acetate buffer (pH 3.6), and 2.5 mL of 20 mM FeCl_3_·6H_2_O, were mixed and incubated for 30 min. This mixed solution is called the FRAP solution. Next, 1 mL of the sample or standard solution (ascorbic acid) was mixed with 3 mL of FRAP solution and incubated for 30 min in the dark. The formation of the colored product, the ferrous tripyridyltriazine complex, was measured at a wavelength of 593 nm. Activity was expressed as mg ascorbic acid equivalent antioxidant capacity (mg AEAC/g).

### 2.13. Antimicrobial Activity Evaluation

The antibacterial properties of *terasi* were assessed using the agar well diffusion method, as evidenced by the presence of inhibition zones [20]. *Staphylococcus aureus* ATCC 25923 and ATCC 25922 were used as the test microorganisms for this evaluation. Initially, bacterial cultures were inoculated into Nutrient Broth (NB; Oxoid, Basingstoke, UK) medium and incubated for 24–48 h. The cultures were then standardized to the turbidity of a McFarland II standard, which was approximately 6 × 10^8^ CFU/mL. Nutrient agar plates were then inoculated with 1 mL of the standardized bacterial suspension. Filter paper discs infused with 20 µL of *terasi* diluted in ethanol (1:10, *w*/*v*) were placed on the agar surface. The plates were then incubated at 37 °C for 24 h, after which the inhibition zone diameters were measured to determine antibacterial activity. Chloramphenicol (Oxoid, Basingstoke, UK) served as the positive control, and distilled water was used as the negative control to ensure assay validity.

### 2.14. Inhibition of α-Amylase Enzyme Activity

The inhibitory activity of *terasi* against the α-amylase enzyme (Sigma-Aldrich, Darmstadt, Germany) was evaluated as described previously [22]. Working solutions were prepared at concentrations of 100, 250, 500, 750, and 100 µg/mL. Each solution (500 µL) was combined with 500 µL of sodium phosphate buffer (0.02 M, pH 6.9, containing 0.006 M sodium chloride) and an α-amylase solution (0.5 mg/mL). The mixture was stored for 10 min at 25 °C. Each tube received 500 µL of a 10 g/L starch solution prepared in 0.02 M sodium phosphate buffer (pH 6.9) containing 0.006 M sodium chloride (Merck, Darmstadt, Germany). The reaction mixtures were then incubated at 25 °C for 10 min. To halt the reaction, 1.0 mL of 3,5-dinitrosalicylic acid reagent was added, followed by heating in a water bath for 5 min and allowing the mixture to cool to room temperature. Absorbance was measured at 540 nm using a spectrophotometer (Thermo Fisher, Waltham, MA, USA). The percentage of α-amylase inhibition was calculated using the following formula: [Abs_blank_ − Abs_sample_]/Abs_blank_ × 100. The sample concentrations and their corresponding percent inhibitions were plotted, and a linear regression equation was derived. The IC_50_ value, which denotes the extract concentration needed to inhibit 50% of α-amylase activity, was calculated based on the *y*-intercept of the regression line, whereas the slope represented the tested extract concentrations.

### 2.15. Inhibition of α-Glucosidase Enzyme Activity

The α-glucosidase inhibitory activity of *terasi* was assessed using a previously described method with minor modifications [23]. A reaction mixture was prepared by combining 10 µL of α-glucosidase enzyme solution (Sigma-Aldrich, Darmstadt, Germany; 1 U/mL), 50 µL of phosphate buffer (100 mM, pH 6.8), and a serial concentration of *terasi* sample (0 to 100 mg/mL). The mixture was incubated for 15 min at 37 °C. Then, 20 µL of 4-nitrophenyl-α-d-glucopyranoside (5 mM) was added, and the mixture was incubated at 37 °C for another 20 min. The reaction was terminated by adding 50 µL of 0.1 M sodium carbonate (Na_2_CO_3_). The absorbance of the reaction mixture was recorded at 405 nm, and the α-glucosidase inhibition percentage was determined using the following formula: 1 − [Abs_A_ − Abs_B_] × 100, where Abs_A_ is the absorbance of the sample and Abs_B_ is the absorbance of the control (phosphate buffer). The IC_50_ values, which indicate the concentration of the sample required to inhibit 50% of the α-glucosidase activity, were determined according to the procedure described for the α-amylase assay.

### 2.16. Data Analysis

Statistical analyses were performed using two-way ANOVA, followed by Duncan’s post hoc test. The analyses were carried out using SPSS version 17 (IBM Corp., Armonk, NY, USA), and a *p*-value of less than 0.05 was regarded as statistically significant. Additionally, an independent *t*-test was employed to assess differences between the two groups, with *p* < 0.05 set as the threshold for statistical significance.

## 3. Results and Discussion

### 3.1. Chemical Properties of Terasi

**Figure 1:**
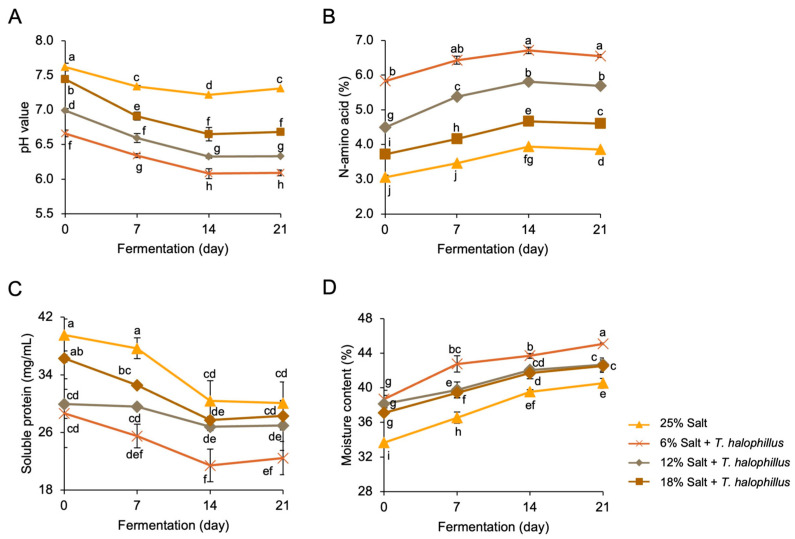
Changes in the chemical properties of *terasi* with and without inoculation of *T. halophilus* at different salt concentrations and during different fermentation stages. (**A**) pH, (**B**) N-amino acid content, (**C**) soluble protein content, and (**D**) moisture content. Different letters indicate statistically significant differences (*p* < 0.05), as determined by the Duncan’s multiple range test.

The pH, N-amino acid, soluble protein, and moisture content of *terasi* inoculated with or without *T. halophilus* at different fermentation stages are shown in Figure 1. The interaction between salt concentration and fermentation duration significantly affected all parameters. The pH of *terasi* ranged from 6.09 to 7.62, with the highest value observed in the control at 0 days of fermentation and the lowest in samples fermented for 21 days with 6% salt. This is in line with previous findings that reported pH values for shrimp paste ranging from 6.94 to 8.31 [24]. Our findings showed that the pH of *terasi* is primarily determined by the salt concentration and fermentation time. Higher salt concentrations inhibit LAB activity, reducing lactic acid production and resulting in higher pH values. Conversely, lower salt concentrations stimulate LAB growth, promoting acid production and lowering pH [25]. During fermentation, the pH gradually decreased owing to organic acid production by LAB, reaching its lowest value on day 14. By day 21, the pH stabilized or slightly increased, probably due to reduced LAB activity and a decrease in lactic acid accumulation. Inoculation with *T. halophilus* led to a more pronounced decrease in pH than that in the control samples. This effect is attributed to the ability of the bacterium to accelerate carbohydrate metabolism and lactic acid production, thereby increasing acidification. The resulting low pH creates an environment unsuitable for the growth of pathogens and spoilage organisms, consistent with findings reported by previous studies [26,27].

The N-amino acid content ranged from 3.06% to 6.85%, with the highest levels found in samples fermented for 21 days with 6% salt, while the lowest moisture content (33.65%) was observed in the control at 0 days of fermentation. These findings are consistent with those of Daroonpunt et al. [24], who reported N-amino acid levels of 2.87–6.85%. The concentration of N-amino acids in *terasi* is influenced by the salt levels and fermentation time. A previous study demonstrated that high salt concentrations inhibit protein hydrolysis by limiting enzymatic activity [28]. During fermentation, LAB proteolytic enzymes break down proteins into peptides and free amino acids, resulting in peak N-amino acid levels by day 14. However, these levels declined by day 21, which might be due to reduced LAB populations and enzyme production. *Terasi* inoculated with *T. halophilus* exhibits higher N-amino acid levels than the control, as *T. halophilus* produces more proteolytic enzymes, accelerating protein hydrolysis [9]. In contrast, the spontaneous fermentation process in the control samples resulted in less selective LAB growth, leading to reduced proteolytic activity [29].

The soluble protein content ranged from 21.10 to 39.53 mg/mL. The lowest levels were found in *terasi* fermented for 14 days with 6% salt, while the highest levels were found in the control at 0 days. These results are in line with previous findings that have reported soluble protein levels in shrimp paste [30]. Salt concentration and fermentation time significantly affected the soluble protein content in *terasi*. High salt concentrations stabilize protein structures by forming a protective hydration layer around the proteins, thereby shielding the peptide bonds from enzymatic hydrolysis [31,32]. During the early stages of fermentation, soluble protein levels decrease as protease enzymes hydrolyze proteins into smaller peptides and amino acids [30]. However, by day 21, soluble protein levels increased again, likely due to reduced LAB activity and a concomitant decline in protease production. Inoculated *terasi* demonstrated higher soluble protein levels than the control, as *T. halophilus* produces robust proteolytic systems that efficiently hydrolyze proteins into amino acids and peptides [33,34].

The moisture content varied from 33.65% to 45.08%, with the highest value found in *terasi* fermented for 21 days at 6% salt and the lowest value found in the control at 0 days, which is consistent with a previous study that reported moisture content of shrimp paste between 33% and 52% [24]. The moisture content of *terasi* is influenced by the interplay between salt concentration and fermentation time. High salt concentrations result in lower moisture content due to the ionization of Na^+^ and Cl^−^ ions, which bind to water molecules and reduce their availability [35]. In contrast, lower salt concentrations during fermentation create osmotic imbalances, prompting LAB to extract water through osmosis [36]. Extended fermentation increases the moisture content, as the acidic conditions promote water release, and prolonged interactions between salt and water enhance water retention in the final product [37]. *Terasi* inoculated with *T. halophilus* retains higher moisture content compared to the control, as the bacterium can adapt to osmotic pressure changes caused by salt, allowing it to survive osmotic stress and retain more water [33]. These findings demonstrate the complex interplay between salt concentration, fermentation time, and bacterial inoculation in shaping the final quality of *terasi*. Understanding these interactions provides valuable insights for optimizing *terasi* production to achieve desired physicochemical properties.

### 3.2. Physical Properties of Terasi

**Figure 2:**
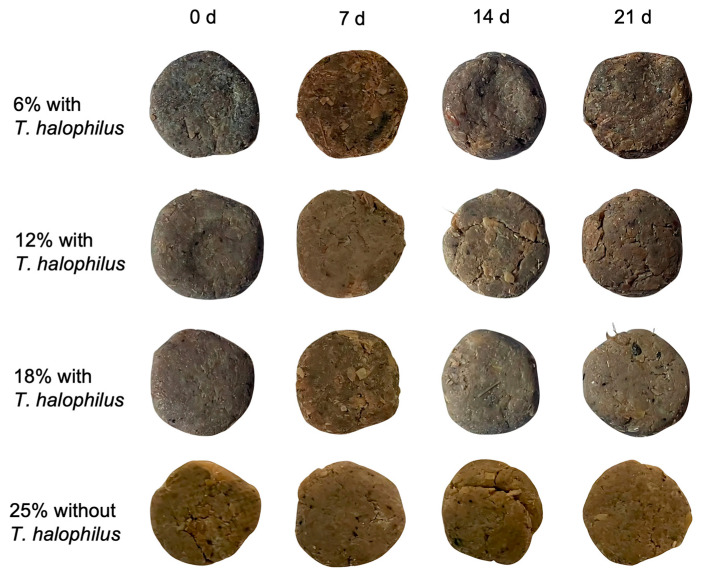
Appearance of *terasi* inoculated with and without *T. halophilus* at different salt concentrations and fermentation times.

The color parameters of *terasi* inoculated with and without *T. halophilus* at different salt concentrations are shown in Table 1 and Figure 2. The color assessment of *terasi* was conducted based on the L*, a*, and b* parameters of the CIE color system. The L* parameter signifies the brightness level, whereas a* reflects the intensity of red or green tones, and b* represents the extent of yellow or blue hues. An increase in the a* and b* values indicates enhanced redness and yellowness, respectively. The interaction between salt concentration and fermentation duration significantly influenced the L*, a*, and b* values. The non-inoculated *terasi* fermented for 7 d displayed the highest L* (52.92 ± 0.007) and b* (27.53 ± 0.063) values. The lowest L* was obtained in *terasi* inoculated with *T. halophilus* with 6% salt addition for 21 d (21.74 ± 0.275), which also yielded the highest a* (10.89 ± 0.098) and ∆*E* (33.55 ± 0.27). Meanwhile, the lowest a* (0.81 ± 0.155) and the highest ∆C* (3.08 ± 0.04) were observed in non-inoculated *terasi* fermented for 0 d. Additionally, the lowest ∆C* (−12.53 ± 0.30) was observed in *terasi* inoculated with *T. halophilus* with 6% salt addition for 0 days.

The non-inoculated *terasi* showed a lighter color than the *terasi* subjected to inoculation at the corresponding fermentation stages, with a noticeable reduction in lightness (L*) as fermentation time increased (Figure 2). The enzymatic activities occurring during *terasi* fermentation can be affected by various factors, such as the composition of raw materials, physicochemical interactions, storage environment, and the presence of specific proteolytic bacteria [38]. For instance, salt interacts with *rebon* shrimp proteins, leading to their breakdown and contributing to *terasi* darkening. Additionally, low salt concentrations are associated with decreased color intensity during fermentation. The formation of low-molecular-weight compounds from fish protein and high-molecular-weight melanoidins during fermentation contributes to increased color intensity [39]. A previous study demonstrated the decline in L* values with fermentation time is attributed to protein and lipid degradation, resulting in a less bright final product [40]. Throughout the fermentation process, the Δ*E* and ΔC* values tended to increase, whereas the L* values decreased. Inoculant addition was found to lower L* and enhance Δ*E*, although it reduced ΔC*. Furthermore, previous studies have demonstrated that the degradation of shrimp lipoproteins during fermentation releases free astaxanthin, a pigment responsible for the red coloration of shrimp paste and recognized for its antioxidant properties [41]. Consequently, the introduction of starter inoculant promotes the development of a browner coloration in *terasi*.

**Figure 3:**
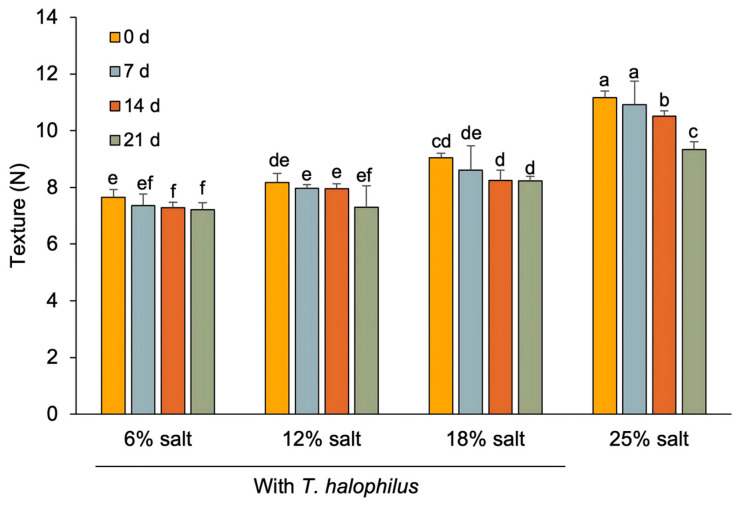
Changes in the texture properties of *terasi* with and without inoculation of *T. halophilus* at different salt concentrations and during different fermentation stages. Different letters indicate statistically significant differences (*p* < 0.05), as determined by the Duncan’s multiple range test.

The interaction between salt concentration and fermentation duration significantly influenced the texture, especially the hardness of *terasi* (Figure 3). The hardness values ranged from 7.22 to 11.17 N, with the highest level observed in non-inoculated *terasi* at the initial fermentation stage (day 0), while the lowest value was recorded in *terasi* inoculated with *T. halophilus* at 6% salt concentration after 21 days of fermentation. An increase in salt concentration corresponded to a harder *terasi* texture. Salt incorporation may enhance *terasi* texture by promoting protein aggregation, improving water retention, and supporting stable gel formation. Salt increases ionic strength, which plays a crucial role in the aggregation behavior of myofibrillar proteins. This aggregation contributes to the formation of a denser and more organized gel network, thereby directly improving the texture of shrimp-based products, such as *terasi*. Salt also facilitates the extraction and dissolution of myofibrillar proteins from shrimp muscle, which is essential for forming a strong and cohesive gel matrix, further contributing to improved texture [42]. This finding aligns with previous studies indicating that salt levels (10–30%) in fermented fish products facilitate the breakdown and extraction of myofibrillar proteins, including actin and myosin, which play a key role in gel formation and binding within restructured meat products [43].

Conversely, prolonged fermentation reduced the hardness of *terasi*, potentially due to the gelation processes. Zang et al. [2] described gelation as the formation of a gel matrix from fish meat proteins, which significantly impacts the texture of fish products. The hardness values observed in this study likely resulted from protein denaturation and gelation of fish muscle proteins. This is in line with previous studies that reported that during the initial stages of fermentation, fish meat undergoes gelation induced by the acidic environment created by microbial activity. As fermentation progresses, hydrophobic interactions contribute to gel network formation in fermented carp meat. However, further reductions in pH due to microbial activity create increasingly acidic conditions, disrupting the gelation process and subsequently softening the final product’s texture [44].

### 3.3. Microbiological Properties of Terasi

**Figure 4:**
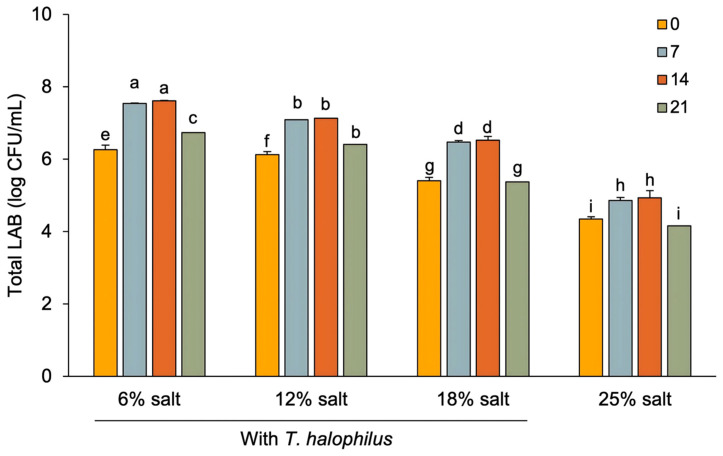
The changes in total lactic acid bacteria in *terasi* with and without inoculation of *T. halophilus* at different salt concentrations and fermentation stages. Different letters indicate statistically significant differences (*p* < 0.05), as determined by the Duncan’s multiple range test.

Salt concentration and fermentation time showed a significant interaction, and both factors individually influenced the total LAB count in *terasi* (Figure 4). The LAB count in the final *terasi* product ranged from 4.35 to 7.61 log CFU/mL, with the highest count observed in *terasi* treated with *T. halophilus* at 18% salt concentration and 14 days of fermentation. Conversely, the lowest LAB count was found in *terasi* without *T. halophilus* at 25% salt concentration and 0 days of fermentation. These results are consistent with previous studies that reported LAB counts in *terasi* products ranging from 4.10 to 6.90 log CFU/mL [1].

The data showed that lower salt concentrations and longer fermentation times (up to 14 days) resulted in higher LAB counts, whereas fermentation beyond 14 days resulted in a decrease. The addition of *T. halophilus* culture increased LAB counts compared to the control (non-inoculated) *terasi*. High salt concentrations have been shown to suppress LAB growth due to increased osmotic pressure, which can damage bacterial cell membranes. Excessive salt disrupts bacterial growth by altering membrane permeability and increasing osmotic stress, which can lead to LAB cell death [45]. During fermentation, LAB counts initially increased; however, by day 21, LAB counts decreased, likely due to nutrient depletion, accumulation of lactic acid metabolites, and increasingly acidic conditions that created an unfavorable environment for LAB survival. LAB mortality under conditions of nutrient depletion, accumulation of metabolic acids, and lack of energy reserves [46]. Non-inoculated *terasi* showed the lowest LAB counts due to spontaneous fermentation and a diverse microbial population, including spoilage organisms [47]. In contrast, inoculation with *T. halophilus* led to controlled fermentation, enhanced lactic acid production, and selective LAB growth, as observed in other studies [30]. Controlled fermentation facilitates more efficient and targeted microbial development than spontaneous fermentation.

### 3.4. Sensory Properties of Terasi

A sensory evaluation was carried out to identify the treatment that yielded the *terasi* most favored by consumers, considering attributes such as color, aroma, and texture. The results revealed that the *terasi* prepared with the addition of bacterial inoculants was consistently favored by the panelists (Table 2). Prolonged fermentation times improved the sensory scores across all attributes. Inoculation with *T. halophilus* significantly enhanced the sensory properties, with the highest scores observed after 14 and 21 days of fermentation. In contrast, salt concentration had varying effects, with lower salt levels yielding the most preferred colors and aromas. The highest color and aroma scores were recorded for *terasi* inoculated with *T. halophilus* and prepared with 6% salt for 14 days (4.56 ± 0.583) and 7 days (4.52 ± 0.509), respectively. The most preferred texture was associated with *terasi* inoculated with *T. halophilus* and prepared with 18% salt for 14 and 21 days (4.04 ± 0.789). Glutamic acid, a precursor to monosodium glutamate and a key contributor to umami flavor, was enhanced by the addition of bacterial inoculants [48]. Similarly, previous studies have reported that lower salt concentrations improve the flavor profile of shrimp pastes [49]. Consequently, inoculant addition not only improved the sensory attributes but also increased consumer acceptability. These findings highlight that bacterial starters can enhance both the quality and consumer preference of *terasi.*

### 3.5. The Optimal Treatment of Terasi Formulation

The optimal treatment was chosen by means of the TOPSIS method in accordance with the steps described in the method section. The initial decision matrix was constructed and subsequently normalized (Table S1), with attribute weights determined using the best–worst method. The corresponding normalized-weighted matrix is provided in Table S2. Positive Ideal Solutions (PIS) and Negative Ideal Solutions (NIS) for each attribute were computed and are presented in Table 3. The Euclidean distances for each alternative were used to compute performance scores (Table 4), which indicate the closeness of each alternative to the PIS. The rankings of the formulations were based on these performance scores, with TOPSIS identifying *terasi* inoculated with *T. halophilus*, containing 6% salt, and fermented for 7 days as the most optimal sample. This formulation had a performance score value of 0.611, the shortest Euclidean distance to the PIS (0.075), and the largest distance from the NIS (0.118). Our results showed that the use of halophilic bacteria as a starter can reduce the fermentation time of shrimp paste, which is consistent with a previous study [5]. The amino acid profile and bioactivity were then further characterized under these optimal conditions.

### 3.6. Amino Acid Profile of Terasi

The amino acid profile of *terasi* inoculated with *T. halophilus*, containing 6% salt, and fermented for 7 days was analyzed and is presented in Table 5. The amino acid content ranged from 6.55 to 86.48 mg/g, with L-glutamic acid, L-aspartic acid, L-leucine, L-alanine, and L-lysine being the predominant components. These findings align with previous studies on low-salt fermented shrimp paste, which identified aspartate, glutamate, alanine, leucine, lysine, arginine, and proline as the dominant amino acids after 30 days of fermentation. However, the amino acid levels observed in the study by Cai et al. [49] were lower (1.59–26.9 mg/g) than in our study, likely due to differences in the raw material, which was wild marine shrimp (*Acetes chinensis*).

### 3.7. Antioxidant Activity of Terasi

The antioxidant activity of *terasi* inoculated with *T. halophilus*, containing 6% salt and fermented for 7 days, was measured and is presented in Table 6. The *terasi* exhibited DPPH radical scavenging activities of approximately 3.904 mg AEAC/g and 8.76 ± 0.22 mg AEAC/g, respectively. Previous studies have reported similar antioxidant properties in fermented shrimp products. For instance, the water-soluble fraction of *Ka-pi*, a traditional Thai fermented shrimp paste, demonstrated DPPH and ABTS radical-scavenging activity as well as ferric reducing antioxidant power (FRAP) in a concentration-dependent manner [21]. The antioxidant activity in fermented protein products is often attributed to bioactive peptides produced during fermentation. According to Najafiam and Babji [50], two newly identified peptides, Leu-Asp-Asp-Pro-Val-Phe-Ile-His and Val-Ala-Ala-Gly-Arg-Thr-Asp-Ala-Gly-Val-His, play a significant role in the antioxidant properties of fermented anchovy fish (*Budu*) extract. It has been postulated that the hydrophobic amino acids in fish peptide extracts are responsible for these effects [51]. These findings suggest that bioactive peptides in *terasi* inoculated with *T. halophilus* may similarly contribute to its antioxidant activity, warranting further investigation to confirm and characterize these bioactive compounds.

### 3.8. Antimicrobial Activity of Terasi

The antibacterial properties of *terasi* were evaluated based on its capacity to suppress the growth of *E. coli* and *S. aureus*, using chloramphenicol as the positive control and distilled water as the negative control. *Terasi* (1 g/10 mL) inoculated with *T. halophilus*, containing 6% salt, and fermented for 7 days, demonstrated significant activity against both *E. coli* and *S. aureus* (Table 6). The inhibition zone diameters of *terasi* inoculated with *T. halophilus* were 32.78 mm for *E. coli* and 30.85 mm for *S. aureus*, which were notably larger than those observed for traditional *terasi* undergoing spontaneous fermentation. Jamilah et al. [52] previously reported inhibition zones of 23.5 mm for *E. coli* and 22.4 mm for *S. aureus* in spontaneously fermented *terasi*. The antimicrobial activity of *T. halophilus*-inoculated *terasi* can be attributed to the antimicrobial metabolites produced by *T. halophilus*. Chikindas et al. [53] reported that *T. halophilus* produces a range of antimicrobial compounds, including organic acids, phenyl lactic acid, organic antimicrobials, hydrogen peroxide (H_2_O_2_), and bacteriocins. Additionally, the 6% salt content in the *terasi* may have contributed to the inhibition zone size by limiting bacterial growth through osmotic pressure [45]. These findings underscore the potential of *T. halophilus*-inoculated *terasi* as a functional food with enhanced antimicrobial properties. Although these results highlight the potential of *T. halophilus*-inoculated *terasi* as a functional food with enhanced antimicrobial properties, the specific compounds responsible for the antibacterial activity remain unidentified. The mechanisms by which these metabolites inhibit different bacterial strains are also not fully understood, warranting further investigation.

### 3.9. Antidiabetic Activity (α-Amylase and α-Glucosidase Inhibition) of Terasi

The anti-diabetic potential of *terasi* inoculated with *T. halophilus*, containing 6% salt and fermented for 7 days, was evaluated using in vitro models to measure its ability to inhibit α-amylase and α-glucosidase enzymes (Table 6). Positioned on the brush border membrane of intestinal cells, these enzymes are essential for carbohydrate digestion and absorption, ultimately contributing to elevated blood glucose levels [54]. The primary objective of inhibiting α-amylase and α-glucosidase is to decelerate the conversion of polysaccharides into absorbable monosaccharides, thereby mitigating postprandial hyperglycemia. Our study demonstrated that *terasi* effectively inhibited the activity of both enzymes, with IC_50_ values of approximately 1.95 mg/mL for α-amylase and 7.24 mg/mL for α-glucosidase. In comparison, acarbose, used as a positive control, exhibited IC_50_ values of 1.52 mg/mL and 0.01 mg/mL for α-amylase and α-glucosidase, respectively. The inhibitory activity of *terasi* inoculated with *T. halophilus* may be attributed to metabolites produced during fermentation. Previous studies have shown that *T. halophilus* produces exopolysaccharides with strong antioxidant potential [55]. Exopolysaccharides from specific microorganisms have also demonstrated the ability to inhibit α-glucosidase activity [56]. Additionally, other metabolites such as bioactive peptides can interfere with glucose metabolism by inhibiting key enzymes through competitive binding involving van der Waals forces, hydrogen bonding, and hydrophobic interactions [57]. Peptides like GLLGY from fermented rice bran and several peptides from *Andrias davidianus* have been shown to effectively suppress both the activities of α-glucosidase and α-amylase [58,59]. Although these results suggest that *terasi* has promising antidiabetic properties, the specific bioactive compounds that cause this inhibitory effect in our samples remain unknown. A synergistic effect of various fermentation-derived metabolites, including peptides and exopolysaccharides, likely contributed to the observed enzyme inhibition. Further investigations are necessary to isolate, identify, and characterize the active compounds and clarify the underlying mechanisms responsible for the antidiabetic activity of *T. halophilus*-inoculated *terasi*.

## 4. Conclusions

This study demonstrated that the inoculation of traditional Indonesian *terasi* with *T. halophilus*, coupled with a reduction in salt concentration and controlled fermentation, markedly enhanced its quality and functionality. The optimized formulation, consisting of 6% salt and a 7-day fermentation period with *T. halophilus*, improved sensory acceptance and physicochemical properties while also demonstrating promising bioactive potential, including antioxidant, antidiabetic, and antimicrobial activities. These findings indicate that targeted microbial inoculation is an effective strategy for developing healthier, functionally enhanced fermented seafood products. Nonetheless, the specific bioactive compounds responsible for these functional properties remain unidentified, and their mechanisms of action are not yet understood. Consequently, further research is imperative to isolate and characterize these compounds, elucidate their mechanisms, and evaluate their safety and efficacy through in vitro, in vivo, and clinical studies. Such investigations will facilitate the development of *terasi* as a scientifically validated functional food with potential health benefits.

## Figures and Tables

**Table 1:** Color of *terasi* with and without *T. halophilus* inoculation at different salt concentrations and fermentation stages.

Treatment	Fermentation Time (Day)	L*	a*	b*	∆ *E*	∆ C*
6% salt with *T. halophilus*	0	26.17 ± 0.084 ^n^	8.23 ± 0.000 ^e^	9.54 ± 0.091 ^k^	30.16 ± 0.51 ^c^	−12.53 ± 0.30 ^k^
	7	25.33 ± 0.063 ^m^	10.32 ± 0.021 ^c^	10.65 ± 0.035 ^j^	30.89 ± 0.76 ^bc^	−10.30 ± 0.14 ^j^
	14	22.43 ± 0.155 ^o^	10.50 ± 0.035 ^b^	11.90 ± 0.056 ^i^	32.87 ± 0.49 ^ab^	−9.26 ± 0.20 ^i^
	21	21.74 ± 0.275 ^o^	10.89 ± 0.098 ^a^	11.99 ± 0.084 ^i^	33.55 ± 0.27 ^a^	−8.94 ± 0.02 ^i^
12% salt with *T. halophilus*	0	47.88 ± 0.141 ^d^	3.17 ± 0.021 ^j^	18.69 ± 0.106 ^h^	7.46 ± 0.85 ^g^	−6.18 ± 0.36 ^h^
	7	47.71 ± 0.155 ^b^	5.40 ± 0.007 ^g^	18.97 ± 0.063 ^g^	8.28 ± 0.48 ^g^	−5.41 ± 0.07 ^g^
	14	44.56 ± 0.091 ^c^	6.81 ± 0.021 ^e^	18.93 ± 0.049 ^g^	10.69 ± 0.25 ^f^	−5.02 ± 0.04 ^f^
	21	32.09 ± 0.070 ^k^	8.70 ± 0.063 ^d^	20.41 ± 0.007 ^e^	20.99 ± 2.41 ^d^	−2.95 ± 0.31 ^d^
18% salt with *T. halophilus*	0	50.69 ± 0.077 ^b^	2.17 ± 0.127 ^k^	18.72 ± 0.028 ^g^	6.45 ± 0.00 ^gh^	−6.29 ± 0.03 ^h^
	7	50.20 ± 0.063 ^b^	3.98 ± 0.091 ^i^	19.99 ± 0.014 ^f^	6.00 ± 2.57 ^gh^	−4.75 ± 0.09 ^f^
	14	46.17 ± 0.000 ^j^	5.32 ± 0.021 ^g^	20.88 ± 0.000 ^e^	7.80 ± 0.25 ^g^	−3.59 ± 0.01 ^e^
	21	35.64 ± 0.106 ^g^	8.10 ± 0.060 ^o^	20.91 ± 0.035 ^e^	17.47 ± 0.80 ^e^	−2.71 ± 0.33 ^d^
25% salt without *T. halophilus*	0	50.97 ± 0.098 ^b^	0.81 ± 0.155 ^m^	25.02 ± 0.106 ^d^	0.66 ± 0.00 ^i^	−0.10 ± 0.14 ^c^
	7	52.92 ± 0.007 ^a^	1.21 ± 0.000 ^l^	25.21 ± 0.000 ^c^	2.44 ± 2.57 ^i^	0.11 ± 0.06 ^c^
	14	49.90 ± 0.077 ^f^	5.18 ± 0.035 ^h^	26.68 ± 0.021 ^b^	4.84 ± 0.25 ^h^	2.04 ± 0.08 ^b^
	21	36.47 ± 0.162 ^e^	6.19 ± 0.021 ^f^	27.53 ± 0.063 ^a^	15.67 ± 0.80 ^e^	3.08 ± 0.04 ^a^

Different letters indicate statistically significant differences (*p* < 0.05), as determined by the Duncan’s multiple range test.

**Table 2:** Sensory properties of *terasi* with and without *T. halophilus* inoculation at different salt concentrations and fermentation stages.

Treatment	Fermentation Time (Day)	Color	Aroma	Texture
6% salt with *T. halophilus*	0	1.68 ± 0.627 ^ab^	1.96 ± 0.611 ^a^	1.76 ± 0.597 ^ab^
	7	4.48 ± 0.585 ^hi^	4.52 ± 0.509 ^g^	3.64 ± 0.637 ^gh^
	14	4.56 ± 0.583 ^hi^	4.48 ± 0.509 ^g^	3.56 ± 0.650 ^de^
	21	4.64 ± 0.860 ^i^	4.44 ± 0.506 ^g^	3.64 ± 0.637 ^hi^
12% salt with *T. halophilus*	0	1.88 ± 0.665 ^b^	2.08 ± 0.862 ^a^	1.64 ± 0.700 ^ab^
	7	2.88 ± 0.665 ^d^	3.28 ± 0.842 ^e^	3.16 ± 0.800 ^de^
	14	3.65 ± 0.820 ^e^	3.36 ± 0.637 ^e^	3.04 ± 0.734 ^de^
	21	4.20 ± 0.707 ^gh^	3.80 ± 0.707 ^f^	3.28 ± 0.678 ^fg^
18% salt with *T. halophilus*	0	2.32 ± 0.748 ^c^	2.56 ± 0.768 ^b^	2.80 ± 0.816 ^d^
	7	2.72 ± 0.791 ^bc^	3.32 ± 0.476 ^e^	3.96 ± 0.789 ^hi^
	14	3.80 ± 0.763 ^fg^	3.76 ± 0.663 ^f^	4.04 ± 0.789 ^i^
	21	4.16 ± 0.800 ^gh^	4.24 ± 0.663 ^g^	4.04 ± 0.789 ^i^
25% salt without *T. halophilus*	0	1.44 ± 0.506 ^a^	2.36 ± 0.757 ^ab^	1.40 ± 0.500 ^a^
	7	2.80 ± 0.763 ^d^	2.32 ± 0.852 ^ab^	1.96 ± 0.840 ^bc^
	14	3.08 ± 0.759 ^d^	2.84 ± 0.943 ^cd^	2.12 ± 0.725 ^gh^
	21	3.76 ± 0.723 ^ef^	3.08 ± 0.759 ^de^	2.62 ± 0.646 ^d^

Different letters indicate statistically significant differences (*p* < 0.05), as determined by the Duncan’s multiple range test.

**Table 3:** Positive (A^+^) and negative ideal solution (A^−^) for the criteria.

Criteria	Weight	A^+^	A^−^
Sensory—Color	0.171	0.058	0.018
Sensory—Aroma	0.282	0.083	0.029
Sensory—Texture	0.096	0.035	0.015
Color—L*	0.048	0.077	0.187
Color—a*	0.048	0.003	0.000
Color—b*	0.048	0.048	0.017
Texture	0.048	0.004	0.007
Moisture Content	0.057	0.056	0.075
N-Amino	0.057	0.002	0.001
Soluble Protein	0.096	0.184	0.100
pH	0.018	0.001	0.001
Total Lactic Acid Bacteria	0.032	0.009	0.005

**Table 4:** Final ranking of *terasi* by TOPSIS in different conditions.

Treatment	Fermentation Time (Day)	Rank	CL	di^+^	di^−^
6% salt with *T. halophilus*	0	5	0.533	0.089	0.101
	7	1	0.611	0.075	0.118
	14	3	0.578	0.091	0.125
	21	2	0.596	0.087	0.128
12% salt with *T. halophilus*	0	15	0.285	0.122	0.049
	7	12	0.365	0.109	0.063
	14	14	0.328	0.117	0.057
	21	4	0.568	0.074	0.097
18% salt with *T. halophilus*	0	10	0.413	0.113	0.079
	7	9	0.421	0.110	0.080
	14	7	0.424	0.105	0.077
	21	4	0.568	0.075	0.098
25% salt without *T. halophilus*	0	8	0.422	0.125	0.091
	7	11	0.409	0.122	0.084
	14	13	0.335	0.118	0.060
	21	6	0.531	0.077	0.087

**Table 5:** Amino acids profile of *terasi* inoculated with *T. halophilus* and 6% salt addition for 7 days.

Amino Acid	Composition (mg/g)	Percentage (%)
L-Alanine	37.02	0.084
L-Arginine	12.01	0.027
L-Aspartic acid	52.09	0.119
Glycine	15.44	0.035
L-Glutamic acid	86.48	0.197
L-Histidine	6.55	0.015
L-Isoleucine	16.80	0.038
L-Leucine	49.56	0.113
L-Lysine	40.23	0.092
L-Valine	24.06	0.055
L-Phenylalanine	28.70	0.065
L-Proline	12.69	0.029
L-Serine	15.32	0.035
L-Threonine	19.18	0.044
L-Tyrosine	22.86	0.052

**Table 6:** The antioxidant, antidiabetic, and antimicrobial activities of *terasi* inoculated with *T. halophilus* and 6% salt for 7 days.

Biological Activity	Value
Antioxidant activity	
DPPH radical scavenging activity	3.90 ± 0.04 mg AEAC/g sample
Ferric reducing antioxidant power	8.76 ± 0.22 mg AEAC/g sample
Antidiabetic activity	
IC_50_ of α-amylase enzyme inhibition	
Acarbose	1.52 ± 0.02 ^b^ mg/mL
*Terasi*	1.95 ± 0.02 ^a^ mg/mL
IC_50_ of α-glucosidase enzyme inhibition	
Acarbose	0.01 ± 0.00 ^b^ mg/mL
*Terasi*	7.24 ± 0.21 ^a^ mg/mL
Antimicrobial activity (diameter zone of inhibition)	
Against *E. coli*	
Chloramphenicol (0.146 g/10 mL)	61.54 ± 0.5 mm
Distilled water (10 mL)	0 mm
*Terasi* (1 g/10 mL)	32.78 ± 2.6 mm
Against *S. aureus*	
Chloramphenicol (0.146 g/10 mL)	59.17 ± 0.8 mm
Distilled water 10 mL	0 mm
*Terasi* (1 g/10 mL)	30.85 ± 1.1 mm

Different letters indicate statistically significant differences (*p* < 0.05), as determined by the independent *t*-test.

## Data Availability

The original contributions presented in the study are included in the article/Supplementary Materials. Further inquiries can be directed to the corresponding author.

## Supplementary Materials

The following supporting information can be downloaded at https://www.mdpi.com/article/10.3390/foods14142419/s1: Table S1: Normalized decision-making matrix N. Table S2: Weighted normalized decision matrix.
