# Supplementary material for: Enhancing the Quality of Traditional Indonesian Shrimp Paste (Terasi) Through Tetragenococcus halophilus 54M106-3 Inoculation: Physicochemical, Sensory, and Bioactivity Insights
Source: Foods. 2025 Jul 9;14(14):2419. doi: 10.3390/foods14142419 (PMC12295852; doi:10.3390/foods14142419)
Supplement: Supplementary file 1 [file foods-14-02419-s001.zip › foods-3696979-supplementary.pdf]

Enhancing the Quality of Traditional Indonesian Shrimp Paste (Terasi) Through *Tetragenococcus halophilus* 54M106-3 Inoculation: Physicochemical, Sensory, and Bioactivity Insights

Muhammad Alfid Kurnianto[1], Safrina Isnaini Adirama[1], Xu Wenxi[2], Sri Winarti[1], Dina Mustika Rini[3*]

[1]Food Technology Study Program, Faculty of Engineering & Science, Universitas Pembangunan Nasional Veteran Jawa Timur, Surabaya, 60294, Indonesia; m.alfid.tp@upnjatim.ac.id
[2]College of Food Science and Technology, Wuhan Business University, Wuhan 430056, PR China;wenxi.xu@outlook.com
[3]Graduate School of Integrated Sciences for Life, Hiroshima University, 1-4-4 Kagamiyama, Higashi-Hiroshima, 739-8528, Japan
*Correspondence: dina@hiroshima-u.ac.jp

Table S1. Normalized decision-making matrix N.

Treatment	Fermentation Time (day)	Sensory - Color	Sensory - Aroma	Sensory - Texture	Color - *L**	Color - *a**	Color - *b**	Texture	Moisture Content	N-Amino	Soluble Protein	pH	Total LAB
6% salt with *T. halophillus*	0	0.12	0.13	0.16	1.93	0.05	0.35	0.10	1.12	0.04	1.40	0.06	0.23
	7	0.33	0.27	0.37	1.87	0.06	0.39	0.09	1.24	0.04	1.24	0.05	0.28
	14	0.33	0.26	0.37	1.66	0.06	0.44	0.09	1.26	0.04	1.05	0.05	0.28
	21	0.34	0.27	0.36	1.61	0.07	0.44	0.09	1.30	0.04	1.10	0.05	0.25
12% salt with *T. halophillus*	0	0.14	0.12	0.17	3.54	0.02	0.68	0.10	1.10	0.03	1.46	0.06	0.23
	7	0.21	0.23	0.27	3.53	0.03	0.69	0.10	1.15	0.03	1.44	0.06	0.26
	14	0.27	0.22	0.28	3.29	0.04	0.69	0.10	1.21	0.04	1.11	0.05	0.26
	21	0.31	0.24	0.31	2.37	0.05	0.75	0.09	1.23	0.04	1.32	0.05	0.24
18% salt with *T. halophillus*	0	0.17	0.21	0.21	3.75	0.01	0.69	0.11	1.07	0.02	1.77	0.06	0.20
	7	0.20	0.29	0.27	3.71	0.02	0.73	0.11	1.14	0.02	1.59	0.06	0.24
	14	0.28	0.30	0.31	3.41	0.03	0.76	0.10	1.20	0.03	1.35	0.06	0.24
	21	0.30	0.30	0.35	2.63	0.05	0.77	0.10	1.23	0.03	1.38	0.06	0.20
25% salt without *T. halophillus*	0	0.11	0.10	0.19	3.77	0.00	0.92	0.14	0.97	0.02	1.93	0.06	0.16
	7	0.21	0.14	0.19	3.91	0.01	0.92	0.14	1.06	0.02	1.84	0.06	0.18
	14	0.23	0.16	0.23	3.69	0.03	0.98	0.13	1.14	0.03	1.48	0.06	0.18
	21	0.28	0.19	0.25	2.70	0.04	1.01	0.12	1.17	0.03	1.47	0.06	0.15

Table S2. Weighted normalized decision matrix.

Treatment	Fermentation Time (day)	Sensory - Color	Sensory - Aroma	Sensory - Texture	Color - *L**	Color - *a**	Color - *b**	Texture	Moisture Content	N-Amino	Soluble Protein	pH	Total LAB
6% salt with *T. halophillus*	0	0.021	0.036	0.015	0.092	0.002	0.017	0.005	0.064	0.002	0.134	0.001	0.007
	7	0.056	0.075	0.035	0.089	0.003	0.019	0.004	0.071	0.002	0.119	0.001	0.009
	14	0.057	0.074	0.035	0.079	0.003	0.021	0.004	0.072	0.002	0.100	0.001	0.009
	21	0.058	0.075	0.035	0.077	0.003	0.021	0.004	0.075	0.002	0.105	0.001	0.008
12% salt with *T. halophillus*	0	0.024	0.034	0.016	0.169	0.001	0.033	0.005	0.063	0.002	0.140	0.001	0.007
	7	0.036	0.065	0.026	0.169	0.002	0.033	0.005	0.066	0.002	0.138	0.001	0.008
	14	0.046	0.063	0.026	0.157	0.002	0.033	0.005	0.070	0.002	0.106	0.001	0.008
	21	0.053	0.068	0.030	0.113	0.003	0.036	0.004	0.071	0.002	0.126	0.001	0.008
18% salt with *T. halophillus*	0	0.029	0.058	0.020	0.179	0.001	0.033	0.005	0.061	0.001	0.169	0.001	0.006
	7	0.034	0.082	0.026	0.177	0.001	0.035	0.005	0.065	0.001	0.152	0.001	0.008
	14	0.048	0.083	0.030	0.163	0.002	0.037	0.005	0.069	0.002	0.129	0.001	0.008
	21	0.052	0.083	0.033	0.126	0.002	0.037	0.005	0.070	0.002	0.132	0.001	0.006
25% salt without *T. halophillus*	0	0.018	0.029	0.019	0.180	0.000	0.044	0.007	0.056	0.001	0.184	0.001	0.005
	7	0.035	0.040	0.018	0.187	0.000	0.044	0.007	0.061	0.001	0.176	0.001	0.006
	14	0.039	0.044	0.022	0.176	0.001	0.047	0.006	0.065	0.002	0.142	0.001	0.006
	21	0.047	0.054	0.024	0.129	0.002	0.048	0.006	0.067	0.002	0.140	0.001	0.005
